# Secondary Sulfate Effects?

**DOI:** 10.1289/ehp.10293

**Published:** 2007-11

**Authors:** Thomas Grahame, George M. Hidy

**Affiliations:** U.S. Department of Energy, Washington, DC, E-mail: Thomas.Grahame@HQ.DOE.gov; Envair/Aerochem Placitas, New Mexico

Maynard et al. (2007) related mortality with ambient particulate black carbon (BC) and sulfate (SO_4_). They also associated SO_4_ in Boston, Massachusetts, with “secondary,” distant “coal combustion.” Their correlation of 0.44 between BC and SO_4_, however, suggests the importance of local source(s) of SO_4_, including vehicle exhaust (especially diesel).

Centrally monitored BC poorly characterizes exposure to traffic emissions and results in exposure misestimation, causing distorted or undetected health associations (Ito et al. 2004). Maynard et al. (2007) address this deficiency by geocoding BC estimates to a decedent’s residence; a central monitor characterizes SO_4_ exposure. In their single-pollutant models, BC and SO_4_ are associated with daily mortality, but in multipollutant models, only BC retains significance.

The association of mortality with SO_4_ is inconsistent. In a review of toxicologic studies, Schlesinger and Cassee (2003) suggested that SO_4_ is benign. In an *in vivo* study of PM_2.5_ (particulate matter ≤ 2.5 μm in aero-dynamic diameter) components, Seagrave et al. (2006) found that lung toxicity and inflammation correlated with vehicular but not secondary particles, including SO_4_. However, vehicular emissions are consistently associated with cardiac or other end points (Grahame and Schlesinger 2007). How does secondary SO_4_ cause mortality, if harmful biological mechanisms have not been found? Or could the mortality association with SO_4_ of Maynard et al. (2007) be linked with other local PM sources?

Maynard et al. (2007) cited mortality associations for coal and traffic tracers reported by Laden et al. (2000), but other study findings differ. In a reanalysis, Schwartz (2003) found that their traffic variable remained significant, but their regional “coal” variable became insignificant. We (Grahame and Hidy 2004) noted that among the six cities described by Laden et al. (2000), Boston had the lowest levels of the “coal tracer” (selenium); only Boston had a significant association with an apparent coal source. Similarly, only in Boston was an association with SO_4_ significant. Finding that local residual oil sources emitted over half the Se and SO_4_ in Boston, these authors (Grahame and Hidy 2004) concluded from toxicology that these results represented effects of residual oil emissions.

SO_4_ is elevated near major roadways. Reponen et al. (2003) showed an SO_4_ gradient that declined based on distance from a midwestern freeway; Riediker et al. (2004) found that among highway-related factors, only the “speed changing” factor, which included emissions from accelerating diesel engines, showed elevated SO_4_. These findings are relevant to the monitoring conditions of Maynard et al. (2007). Other Boston studies show commingling between SO_4_ and vehicular indicators. Clarke et al. (2000), for example, reported that loadings of BC are as high in an SO_4_ indicator as in the traffic indicator. Because coal plants emit virtually no BC (Edgerton E, Mueller P, Monroe L, Jansen J, Waid C, unpublished data), high BC in urban SO_4_ indicators reflects both SO_4_ and BC from diesels in an SO_4_ factor, derived from measurements near a thoroughfare. The SO_4_–BC correlation described by Maynard et al. (2007) thus suggests that SO_4_ associations reflect statistical comingling of vehicular and coal emissions.

Association of harm from traffic emissions, but not SO_4_ (or secondary aerosols generally), has been found in panel and epidemiology studies that *a*) precisely measure exposure to local vehicular emissions, and *b*) test effects of secondary PM_2.5_–SO_4_ compared with local emissions. Health end points in such studies include long-term mortality, heart rate variability reduction, ST-segment depression, cardiac effects, vasoconstriction, increased blood pressure, or morbidity [reviewed by Grahame and Schlesinger (2007)]. Kodavanti et al. (2005) found no effects only with the highest SO_4_ concentrations.

We suggest that findings of harmful exposure to secondary SO_4_ per se are tenuous until physiologic mechanisms are identified that support toxicity near ambient concentrations.
